# Molecular Evidence of Emerged Pulmonary Lophomoniasis due to *Lophomonas blattarum* among Hospitalized Patients in Southwestern Iran: A National Registry-Based Study

**DOI:** 10.1155/2022/6292823

**Published:** 2022-05-25

**Authors:** Kobra Mokhtarian, Simin Taghipour, Maryam Nakhaei, Amirmasoud Taheri, Ali Sharifpour, Mahdi Fakhar, Hajar Ziaei Hezarjaribi

**Affiliations:** ^1^Cellular and Molecular Research Center, Basic Health Sciences Institute, Shahrekord University of Medical Sciences, Shahrekord, Iran; ^2^Department of Medical Mycology and Parasitology, School of Medicine, Shahrekord University of Medical Sciences, Shahrekord, Iran; ^3^Toxoplasmosis Research Center, Communicable Diseases Institute, Iranian National Registry Center for Lophomoniasis (INRCL), Mazandaran University of Medical Sciences, Sari, Iran; ^4^Pulmonary and Critical Care Division, Imam Khomeini Hospital, Mazandaran University of Medical Sciences, Sari, Iran

## Abstract

**Objectives:**

*Lophomonas* protozoan is an emerging pathogen transmitted through arthropods such as cockroaches. Lophomoniasis is still a mysterious disease with many unknown epidemiological aspects. The current study aimed to determine the prevalence of lophomoniasis among patients who were hospitalized in Hajar Hospital, Shahrekord, southwestern Iran, using a conventional PCR technique.

**Methods:**

In this retrospective study, 132 frozen bronchoalveolar lavage fluid (BALF) specimens from patients with respiratory disorders hospitalized in Hajar Hospital, Shahrekord district, southwestern Iran, were analyzed during 2020-2021. Samples are referred to the Iranian National Registry Center for Lophomoniasis (INRCL), Mazandaran Province, Northern Iran, for detecting *Lophomonas* spp. infection by a conventionally small subunit ribosomal RNA (SSU rRNA) PCR test.

**Results:**

A total of 132 frozen BALF specimens were examined, 36 (27.3%) tested *Lophomonas* spp. positive using the conventional PCR technique. Also, based on sequencing data and blast analysis, the presence of *L. blattarum* species was confirmed. The average age of *Lophomonas* spp.‐ positive patients was 67.*02* *±* *15*.14 years. Out of the 36 positive subjects, *63.9*% were male and 36.1% female. Male and *Lophomonas* infection had a significant correlation (*p*=0.001). Our findings revealed that *L. blattarum* infected nonsmokers more than smokers (*p*=0.001). The most common underlying disease was also bronchitis

**Conclusion:**

Our results showed, for the first time, that pulmonary lophomoniasis caused by *L. blattarum* is a common and emerging disease in the study area, southwestern Iran. Furthermore, our findings support the use of the PCR test to detect *Lophomonas* infection in archived frozen clinical samples.

## 1. Introduction

*Lophomonas* spp. is an emerging protozoan pathogen that infects the human lungs [1]. This flagellated parasite enters the human body via inhalation cysts which are repelled by reservoir hosts, mostly cockroach feces [2, 3]. Its trophozoites exist in the lower and upper respiratory tracts and cause an infection there. The clinical symptoms are similar to those of other respiratory infections, such as fever, cough, and sputum [4]. Microscopic examination and, more recently, polymerase chain reaction (PCR) techniques are used to diagnose this parasite using a sample taken from the bronchoalveolar lavage fluid (BALF) and sputum [4, 5]. Over the last decade, lophomoniasis has been reported in several Asian countries, whereas, it mostly belongs in Iran and China [1, 4–7]. However, *Lophomonas* has recently been isolated from German cockroaches trapped in some hospitals in Sari, Mazandaran Province, Northern Iran [8]. A registry-based study in the Mazandaran Province also found lophomoniasis in 14% of the patients examined [7]. Moreover, the potential association between *Lophomonas* and chronic respiratory infections has been raised recently, particularly when it can cause cavitary lung lesions [4, 5, 9]. Thus, for the first time, we attempted to determine the prevalence of lophomoniasis among patients who suffered from various respiratory complaints and were hospitalized in Hajar Hospital, Shahrekord, southwestern Iran, using the PCR technique.

## 2. Methods

In this retrospective study, 132 frozen BALF samples were used to detect *Lophomonas*. First, BALF samples were taken from hospitalized patients with various respiratory disorders who were candidates for fiberoptic bronchoscopy (FOB) for several diagnostic workups in Hajar Hospital, Shahrekord, southwestern Iran, throughout 2020–2021. Then, the frozen samples were sent to the Iranian National Registry Center for Lophomoniasis (INRCL) to detect *Lophomonas* DNA by the conventional PCR method. Demographic (age and gender), clinical (underlying diseases), and smoking status data were obtained. The inclusion criteria were patients who are a candidate for bronchoscopy and aged over 18 years old, and also, BALF samples with a volume of less than 2 mL were excluded.

### 2.1. Fiberoptic Bronchoscopy (FOB)

**Figure 1:**
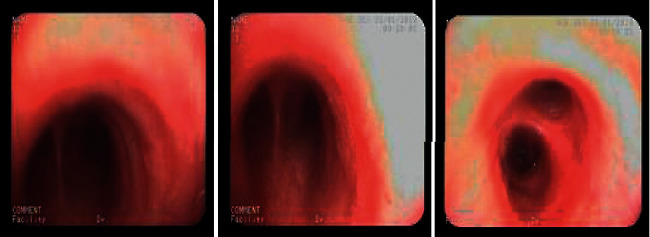
Mild hyperemia in the bronchus intermedius of a patient suffering from chronic cough.

A flexible FOB examination (Figure 1) was done for all patients (*n* = 132) who were candidates for bronchoscopy in a fully sterile condition in the bronchoscopy room or operation room. Wedging the bronchoscope's tip into the nondependent lobes, particularly the middle lobe of the right lung and the lingula of the left lung, provided a BALF specimen. 5–20 mL of sterile normal saline was instilled 2–4 times and divided into 5–20 vials. To extract the saline, gentle manual suction was performed. BALF specimens (approximately 5 mL) were collected in sterilized containers and frozen at -80°C before being transported to the INRCL laboratory in Sari, Northern Iran, for molecular analysis.

### 2.2. DNA Extraction

Genomic DNA was extracted from 132 frozen BALF samples according to Fakhar et al.'s study with slight modifications [5] as follows: After thawing the samples, they were centrifuged at 1200 × *g* for 5 min. About 200 *μ*L of the sediments was mixed with 200 *μ*L of digestive buffer, which consisted of 50 mM Tris–HCl (pH 7.6), 1 mM EDTA, and 1% Tween 20. Following homogenization, 20 *μ*L of proteinase K solution containing 20 mg/mL of enzyme was added and incubated at 55°C for 2 hours. The solution was shaken forcefully and centrifuged at 13,000 × *g* for 15 min after 200 *μ*L of phenol, chloroform, andisoamyl alcohol (25 : 24 : 1) were added. At this point, 200 *μ*L of supernatant was added to 400 *μ*L of cold absolute ethanol inside the microtube, which was then stored at −20°C for 2 hours. Accordingly, 200 *μ*L of 70% ethanol was added to the precipitate and spun. Next, the samples were air-dried and suspended in 50 *μ*L of double distilled water and stored at 4°C until examination. For all that, DNA concentrations were measured using a NanoDrop spectrophotometer (NanoDrop Technologies, Montchanin, DE, USA) and were adjusted to about 20 ng/*μ*L for the PCR assay.

### 2.3. Conventional SSU rRNA- PCR Assay

**Figure 2:**
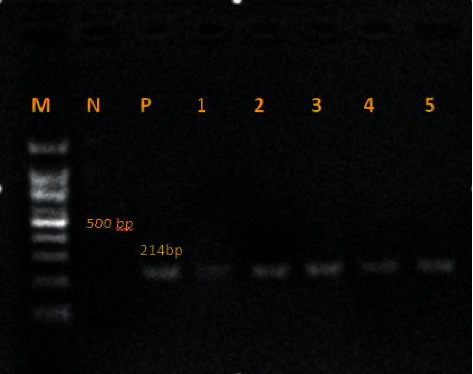
The 214-bp band of the PCR products is shown in 1.5% agarose gel electrophoresis, confirming *Lophomonas* spp. :M 100-bp DNA Marker; N: negative sample (distilled H20); P: positive sample (*L. blattarum*, accession number: MN243135); 1–5: patient specimens of PCR products.

The PCR reaction was set up in a volume of 25 *μ*L, which consisted of 5 *μ*L of the extracted DNA, 12.5 *μ*L of Master Mix (Fermentas Inc.), 1 *μ*L of each primer forward (F) and reverse (R), which was planned from small subunit ribosomal RNA (SSU rRNA), and 5.5 *μ*L of double distilled water [5]. Thirty-five cycles were performed in a thermocycler (Corbett Research, Sydney, Australia) with initial denaturation at 94°C for 2 min, followed by 40 cycles of 94°C for 1 min, 57°C for 1 min, and 72°C for 1 min, and then, a final extension at 72°C for 3 min. Consequently, 6 *μ*L of the PCR products were analyzed on a 1.5% (w/v) agarose gel by electrophoresis in Tris–borate-EDTA (TBE) buffer. A 214-bp band corresponding to *Lophomonas* spp. was observed with UV transillumination after staining with SYBR® Safe Stain (Invitrogen®). For positive control, *Lophomonas blattarum (L. blattarum*) DNA samples (Accession number: MN243135) were used. Sterile distilled H20 was considered a negative control (Figure 2). The Sanger sequencing method and BioEdit software (v.7.2) were used for the sequencing and editing of the amplicons, respectively.

### 2.4. Data Analysis

For qualitative variables, we use percentage and frequency, and for quantitative variables, we use mean and standard deviation. To compare the variables, Chi-square and Fisher's exact tests were used. A *p* value less than 0.05 was regarded as statistically significant. Demographic data were analyzed by IBM SPSS version 26.

## 3. Results

Out of 132 examined patients*, L. blattarum* infection was found in 36 (27.3%) patients. Out of the 36 positive subjects, 23 (63.9%) were male and 13 (36.1%) were female. Male and *Lophomonas* spp. infection had a significant correlation (*p*=0.001). The average age of positive patients was 67.02 ± 15.14 years. There was no statistically significant correlation between age range and *Lophomonas* infection (*p*=0.22). However, we found that almost 83.3% of our patients were aged over 60 years old.

Regarding patients' chief complaints, the most common clinical symptoms among them were chronic cough and dyspnea. Our findings also revealed that *L. blattarum* infection infected nonsmokers more than smokers (*p*=0.001). Moreover, bronchitis was significantly the most common underlying disease (*p*=0.013) (see Table 1).

Also, three high-quality BALF specimens with sharp and nonsmear bands were sequenced to confirm PCR results and deposited in the GenBank by BankIt (Accession Numbers: MZ093077-79). The identity and query coverage of these isolates in comparison with all available Iranian and only Thai *L. blattarum* isolates (Accession Numbers: MN243135-136, MZ093079.1, OL477423.1, OL477422.1, OL477431.1, JX020505.1) were 97–100%.

## 4. Discussion

Our study showed that the prevalence of *L. blattarum* infection was 27.3%, which was greater than the other study in Northern Iran which found a prevalence of 14% [7]. Thus, the results showed the establishment of a new endemic focus in the studied area. Although lophomoniasis is routinely diagnosed by microscopic examination (mostly wet preparation), the morphology of this protozoa is identical to that of the normal or degenerated bronchial epithelial cells such as creola bodies and ciliocytophtoria [4, 10–14]. As a result, discrimination is difficult for an inexperienced microscopist, and in the case of low infection intensity, it could lead to an underdiagnosis or overdiagnosis judgment. The development of a PCR test to detect *Lophomonas* DNA recently by Fakhar et al. may lead to reducing or avoiding these diagnostic pitfalls [5].

Moreover, it should be noted that all BALF samples utilized in this study were frozen, which provides an opportunity to do retrospective investigations to look into various unknown aspects of this emerging *Lophomonas* infection. Other advantages of the PCR method are as follows: detection of the infection in cases of low parasite burden; identification of parasite species; lack of access to fresh clinical specimens; lack of access to cytology smears; overcoming microscopic diagnostic pitfalls; determining the molecular mechanisms of drug resistance in the future [4, 5, 14]. As a result, the PCR-based test appears to offer a convenient and reliable method to detect *Lophomonas* infection in archived samples, particularly in retrospective studies.

The majority of the patients in our study were men, which is in line with previous research findings [2, 14, 15]. This outcome can be interpreted from a variety of perspectives. As we all know [16], men and women are behaviorally and biologically different. These differences can be attributed to men's increased exposure to the outside environment compared to women's exposures [17, 18], as well as testosterone's impact on the majority of parasitic infections [19–21].

Our findings reveal that *Lophomonas* infection is significantly more common in nonsmoking individuals. Accordingly, we hypothesized that smokers are less infected by *L. blattarum* due to damage to the normal epithelium as a parasite attachment site accordingly. However, it is possible that cigarette smoke contains substances that are unfavorable for the survival of the protozoan infecting humans. However, there is evidence that smoking may protect against certain diseases such as sarcoidosis and uterine myoma [22, 23].

All 36 *Lophomonas*-positive patients in this study had chronic respiratory symptoms. 15 (41.6%) individuals suffered from bronchitis, and 9 (16.6%) were hospitalized due to COPD exacerbation. Given the coexistence of these disorders and lophomoniasis, it is clear that this protozoan possibly poses a risk for disease flare-up. However, these preliminary findings are in line with our experience in INRCL, where seven occurrences of asthma exacerbation and *Lophomonas* in children in Northern Iran were found. Further clinical studies are highly suggested regarding the potential association between lophomoniasis and chronic obstructive pulmonary disorders.

In our study, about 30% of the patients suffer from underlying diseases such as diabetes, hypertension, acute nephritic syndrome, myocardial infraction, and hypothyroidism. Underlying diseases were reported at 33% in Yao et al.'s study [24]. Lophomoniasis has been reported in immunosuppressive conditions such as hematopoietic transplantation [25] or leukemia [26] and also in immunocompetent patients [1, 27]. Most of our patients were on a wide range of antibiotics that were not effective. Following confirmation of the *Lophomonas* diagnosis, the patients were treated successfully with metronidazole 500 mg tablets three times a day.

Overall, knowing a lot about the prevalence of *L.blattarum* could help us rescue patients who suffer from chronic respiratory symptoms and save money and supplies that would have been spent on a missed diagnosis. As a final point, more than 80 years after the publication of the first article regarding *Lophomonas*, it still remains unfamiliar to many scientists and physicians worldwide. Further investigations with international collaboration are urgently required to determine the global burden of the enigmatic vector-borne parasitic disease [28] as well as to define the potential vector population.

## 5. Conclusion

Our results showed, for the first time, that *L. blattarum* infection is a common and emerging infection in the study area, southwestern Iran. Furthermore, our findings support the application of PCR to detect *Lophomonas* infection in archived frozen clinical samples. As a whole, the relatively high prevalence of *L.blattarum* in this area could support the establishment of a new endemic focus, and that this could have an impact on the public health burden in the area, which will likely be very helpful for health workers and physicians.

### 5.1. Limitations

First, lack of access to fresh BALF specimens for microscopic examination; second, a relatively small sample size; third, lack of access to patients and follow-up of their treatment process.

## Figures and Tables

**Table 1:** Characteristic of positive *Lophomonas* infection among hospitalized patients in Shahrekord, southwestern Iran.

Characteristic	Frequency (percentage)	*p* value
Gender Male Female	23 (63.9) 13 (36.1)	0.001
Age 31–40 41–50 51–60 61–70 71–80 >81	2 (5.5) 4 (11.1) 5 (13.8) 9 (25) 6 (16.6) 10 (27.7)	0.22
Smoking status Smoker Nonsmoker	11 (30.5) 25 (69.5)	0.001
Underlying diseases and comorbidities Diabetes mellitus Influenza Hypertension Tuberculosis Bronchitis COPD Others ^*∗*^	5 (13.8) 8 (22.2) 4 (11.1) 5 (13.8) 15 (41.6) 9 (16.6) 8 (22.2)	0.013

^*∗*^Others: acute nephritic syndrome, myocardial infraction, and hypothyroidism. COPD: chronic obstructive pulmonary disease.

## Data Availability

The data are available with the corresponding author upon request.
